# Sequence Analysis of a Jumbo Bacteriophage, *Xoo-sp14,* That Infects Xanthomonas oryzae pv. oryzae

**DOI:** 10.1128/MRA.01072-20

**Published:** 2020-11-25

**Authors:** Amina Nazir, Zhaoxia Dong, Jin Liu, Xianglilan Zhang, Rana Adnan Tahir, Neelma Ashraf, Hong Qing, Donghai Peng, Yigang Tong

**Affiliations:** a Key Laboratory of Molecular Medicine and Biotherapy in the Ministry of Industry and Information Technology, Department of Biology, School of Life Sciences, Beijing Institute of Technology, Beijing, China; b State Key Laboratory of Pathogen and Biosecurity, Beijing Institute of Microbiology and Epidemiology, Beijing, China; c State Key Laboratory of Agricultural Microbiology, College of Life Science and Technology, Huazhong Agricultural University, Wuhan, People’s Republic of China; d Industrial Biotechnology Division, National Institute for Biotechnology and Genetic Engineering, Constitute College of Pakistan Institute of Engineering and Applied Sciences, Faisalabad, Pakistan; Queens College

## Abstract

A jumbo bacteriophage, *Xoo-sp14*, infecting Xanthomonas oryzae pv. oryzae was isolated from rice fields in China. Here, we report the complete genome sequence of this phage, revealing that it had a linear double-stranded DNA (dsDNA) molecule 232,104 bp long, with a G+C content of 58%. It has 251 annotated protein-coding sequences.

## Abstract (precis)

A jumbo bacteriophage, *Xoo-sp14*, infecting Xanthomonas oryzae pv. oryzae was isolated from rice fields in China. Here, we report the complete genome sequence of this phage, revealing that it had a linear double-stranded DNA (dsDNA) molecule 232,104 bp in length, with a G+C content of 58%. It has 251 annotated protein-coding sequences.

## ANNOUNCEMENT

Xanthomonas oryzae pv. oryzae is considered one of the most important agents of bacterial blight (BB) on rice (1). This disease creates serious threats to this major crop, leading to destruction and an alarming situation for global food security (2). Bacteriophages are one of the most abundant entities that exist everywhere in the environment and are potent for controlling bacterial diseases (3). Previously, we isolated multiple bacteriophages as potential biocontrol agents against X. oryzae pv. oryzae (4).

In this study, we isolated and characterized a novel *Myoviridae* bacteriophage, *Xoo-sp14*, which infects X. oryzae pv. oryzae strain PXO99A. *Xoo-sp14* was isolated from an agricultural soil (118°35′E, 35°19′N) in Shandong, east China, through the sample enrichment method (4). The soil sample was mixed in sterile water and shaken in order to release the phage particles. The bacteriophage was extracted through a 0.22-μm filter from the mixture. The presence of phage was then confirmed through a spot testing assay with double-layer plates, followed by three single plaque purification steps (4). For phage replication, a filtered medium was incubated with PXO99A in nutrient broth at 28°C with shaking at 220 rpm for 24 h.

Genomic DNA was extracted using a phenol-chloroform protocol (5). The sequencing library of phage was prepared using the New England BioLabs (NEB) Ultra II kit v3. Whole-phage-genome sequencing was performed by Berry Genomics Biotechnology Co., Ltd. (Beijing, China), using Illumina HiSeq 2500 paired-end sequencing technology, with an average read length of 150 bp. The low-quality sequences and short reads were filtered by Trimmomatic software v0.36 (6). SOAP *de novo* version 2.0 was used to assemble the sequences (7). The complete genome of *Xoo-sp14* was annotated by Rapid Annotation using Subsystem Technology (RAST; http://rast.nmpdr.org) (8). All predicted open reading frames (ORFs) were manually double checked by searching against the nonredundant National Center for Biotechnology Information (NCBI) database by using PSI-BLAST (E value = 0.0001). A tRNA scanner (http://lowelab.ucsc.edu/tRNAscan-SE/index.html) was used to predict tRNA genes (9). After the high-throughput sequencing, we got 3,687,722 filtered paired-end reads. All the reads were assembled into a single contig, and the depth of coverage was approximately 2,234×.

*Xoo-sp14* had a linear double-stranded DNA (dsDNA) molecule of 232,104 bp, with a G+C content of 58%. A total of 251 ORFs and no tRNA were found in the genome of *Xoo-sp14*. A total of 13 genes are unknown, and 190 were annotated as hypothetical proteins. However, 48 ORFs were identified as corresponding to putative functional proteins, highlighting the novelty of the phage. Based on putative functions of the ORFs, the *Xoo-sp14* genome was divided into nucleotide metabolism (kinase, dihydrofolate reductase, dCTP deaminase, thymidylate synthase, RNA polymerase beta subunit, and phosphatase), replication (DNA helicase and DNA polymerase), structural components (major capsid protein, major tail protein, minor tail protein, and large terminase subunit), and lysis (cell wall hydrolase, lysozyme, and protease) modules.

Detailed genomic analysis of *Xoo-sp14* will contribute to future insights into the unique structural attributes of jumbo phages and their potential as biocontrol agents.

Default parameters were used for all software used in this study.

### Data availability.

The complete genome sequence of *Xoo-sp14* was submitted to GenBank under the accession number MT939492. The raw sequence reads were deposited to NCBI under the SRA accession number SRR12606159.
